# Imaging-derived neuromuscular ultrasound phenotypes are associated with functional status in amyotrophic lateral sclerosis

**DOI:** 10.1007/s00415-026-13705-4

**Published:** 2026-02-21

**Authors:** Ying Wang, Hao Zhang, Tianhua Yang, Jialei Luo, Ting Lin, Xinyi Yan, Junlin Ding, Yuxuan Qiu, Min Zhao, Gaoyi Yang

**Affiliations:** 1 Department of Ultrasonography, Affiliated Hangzhou First People’s Hospital, School of Medicine, Westlake University, Hangzhou, China; 2 Motor Neuron Disease Diagnosis and Treatment Center/Department of Neurology, Affiliated Hangzhou First People’s Hospital, School of Medicine, Westlake University, Hangzhou, China; 3 Department of Ultrasonography, The Fourth School of Clinical Medicine, Zhejiang Chinese Medical University, Hangzhou First People’s Hospital, Hangzhou, China; 4 Hangzhou Normal University Division of Health Sciences, Hangzhou First People’s Hospital, Hangzhou, China

**Keywords:** Amyotrophic lateral sclerosis, Neuromuscular ultrasound, Unsupervised clustering, Phenotypic heterogeneity, ALSFRS-R

## Abstract

**Background:**

Amyotrophic lateral sclerosis (ALS) presents with marked clinical heterogeneity, complicating diagnosis and management. Neuromuscular ultrasound (NMUS) provides a non-invasive means to visualize peripheral nerve and muscle integrity, but its potential to delineate ALS subtypes has not been systematically explored.

**Objective:**

To identify clinically meaningful ALS subgroups through unsupervised clustering of NMUS features integrated with clinical and electrophysiological data.

**Methods:**

A total of 454 ALS patients (August 2024–December 2025) underwent standardized NMUS assessment, including muscle thickness, echogenicity, and nerve cross-sectional area, alongside ALSFRS-R, manual muscle testing (MMT), and compound muscle action potentials (CMAPs). K-means clustering was applied to standardized NMUS variables, with cluster stability assessed using silhouette coefficients, sensitivity analyses (*k* = 2–5), and resampling-based adjusted Rand indices. Multivariable regression examined associations between cluster membership and ALSFRS-R.

**Results:**

Two reproducible NMUS-based subgroups were identified: a Mild cluster (*n* = 288, 63.4%) and a Severe cluster (*n* = 166, 36.6%). The Severe cluster showed reduced muscle thickness and higher echogenicity across multiple sites, together with lower ALSFRS-R scores (adjusted *β* = − 3.84, 95% CI − 5.41 to − 2.27, *P* < 0.001). Cluster membership correlated negatively with MMT and CMAP amplitudes, supporting functional and electrophysiologic validity. Stability metrics confirmed robustness of the two-cluster solution.

**Conclusion:**

Integrating NMUS with clinical data enables objective, imaging-derived stratification of ALS patients into biologically and functionally distinct subgroups. This approach offers a pragmatic framework for phenotypic characterization and may inform personalized monitoring and trial design in ALS.

**Supplementary Information:**

The online version contains supplementary material available at 10.1007/s00415-026-13705-4.

## Introduction

Amyotrophic lateral sclerosis (ALS) is a progressive neurodegenerative disease characterized by the degeneration of motor neurons in the brain and spinal cord, resulting in muscle weakness, atrophy, and eventual loss of voluntary motor control, ultimately leading to death [1, 2]. Approximately 90% of ALS cases are generally considered sporadic, while around 10% are familial (with some reports suggesting 5–10% familial cases) [3].With a global incidence of approximately 2–3 cases per 100,000 people, ALS represents a significant public health challenge, particularly in aging populations [4, 5]. Multiple population-based studies have demonstrated that the incidence of ALS increases with age, reaching its peak in older adults, especially between 60 and 80 years of age [3]. The median survival for ALS patients is only 3–4 years, underscoring the urgent need for effective diagnostic and therapeutic strategies [6].

ALS patients exhibit a wide range of clinical presentations, with early symptoms varying significantly from person to person. While the diagnosis of ALS is based on well-defined clinical criteria, significant variability in progression patterns, functional disability trajectories, survival rates, and neuropsychological profiles poses significant challenges for developing individualized treatment strategies within the current diagnostic paradigm [7–9]. Considering the notable variability in disease progression and therapeutic responses among ALS patients, subtype classification has emerged as an essential strategy for advancing precision care in ALS management [10, 11]. Importantly, emerging evidence suggests that therapeutic responses may differ substantially across subtypes [12]. For instance, patients with predominantly lower motor neuron involvement may show more favorable responses to interventions aimed at preserving muscle function, whereas those with bulbar-onset ALS often experience limited benefit from such approaches due to rapid involvement of speech and swallowing functions [13]. Similarly, cognitive and behavioral subtypes associated with frontotemporal dysfunction may require integrated neuropsychological and supportive interventions that extend beyond conventional motor-targeted therapies [14]. Subtype analysis has been extensively employed in research on complex diseases, including cancer [15], cardiovascular disorders [16], metabolic disorders [17], and neuropsychiatric conditions [18]. This analytical approach makes a significant contribution to elucidating the underlying mechanisms of complex diseases. It also has demonstrated notable advantages in clinical applications, where it has reduced clinical heterogeneity by stratifying patients into distinct disease categories.

To address the limitations of clinical diagnosis in patients with ALS, cluster analysis has emerged as a powerful tool for integrating multiple data types, including imaging, genetics, and biomarkers, thereby allowing for a more precise identification of distinct ALS subtypes. Cluster analysis, a form of unsupervised machine learning, is highly effective in uncovering hidden patterns and inherent structure within datasets by grouping similar data into clusters [19]. Unlike supervised learning methods, cluster analysis does not rely on prior knowledge or labeled data to define categories, making it particularly useful for exploratory data analysis and discovering underlying relationships within datasets [20]. Previous studies have demonstrated the utility of cluster analysis in ALS research [11, 21]. Radiographically, Bede et al. conducted clustering analysis using only brain MRI data from ALS patients and identified two relatively distinct subtypes with significant clinical features [22].

Neuromuscular ultrasound (NMUS) has demonstrated significant value in assessing muscles and peripheral nerves due to its non-invasive, portable, and cost-effective nature. Previous studies have shown that ultrasound can detect early muscular changes in ALS patients, including alterations in muscle thickness, fascicle length, and muscle stiffness [23, 24]. To the best of our knowledge, no cluster analysis has been conducted on NMUS features; thus, potential clinical subtypes based on NMUS remain unclear.

This study shows that unsupervised clustering of neuromuscular ultrasound features can identify distinct patient subgroups that differ in clinical characteristics. By characterizing phenotypic heterogeneity through nerve cross-sectional area, echogenicity, and fasciculation patterns at a single time point, our approach provides a robust framework for phenotypic stratification. These findings directly inform the development of targeted therapeutic strategies and advance precision medicine in ALS by enabling biologically grounded patient classification rather than relying solely on clinical staging.

## Patients and methods

### Study design and study population

This retrospective study enrolled 454 patients with amyotrophic lateral sclerosis (ALS) who were evaluated at Westlake University School of Medicine Affiliated Hangzhou First People’s Hospital (Hangzhou, Zhejiang Province, China) between August 1, 2024, and December 1, 2025. The study was conducted in accordance with the latest revision of the Declaration of Helsinki [25]. As this study used existing medical records without direct intervention and posed minimal risk to participants, the Institutional Review Board of the First Affiliated Hospital of Westlake University School of Medicine approved a waiver of informed consent (approval no. 2025ZN034-1). All patients met the internationally accepted Gold Coast criteria for ALS [26]. We analyzed demographic characteristics, laboratory indices, and clinical features to characterize phenotypic heterogeneity in this ALS cohort.

### Clinical and laboratory assessment

Demographic and clinical data were collected for each patient, including age, sex, body mass index (BMI), smoking status, and alcohol consumption habits. Clinical information related to ALS was also documented, encompassing disease duration, site of onset, speech function, and graded muscle strength in the upper and lower limbs. Muscle strength was evaluated using manual muscle testing (MMT), a widely used clinical assessment method, with scores ranging from 0 (no muscle contraction) to 5 (normal strength) [27]. On the day of the clinical examination, blood samples were drawn from each patient, and laboratory assessments were conducted using an automated analyzer and included lactate (LAC) levels, platelet (PLT), neutrophil (NEU), monocyte (MONO), and lymphocyte (LYM) counts, and erythrocyte sedimentation rates (ESR). When available, electrophysiological assessments were extracted from the medical records, including nerve conduction study parameters and compound muscle action potentials (CMAPs) from standard target muscles, to provide objective measures of lower motor neuron involvement.

### Patient NMUS data acquisition

#### Patient positioning and preparation

To ensure accurate and consistent ultrasound measurements, all patients were placed in a supine position (lying flat on their back) during the assessment. This positioning is critical because it helps relax the muscles, reducing the likelihood of muscle tension or contraction that could introduce measurement errors [28]. Maintaining a standardized posture minimizes variabilities associated with different body positions, resulting in more reliable and reproducible results.

#### NMUS protocol

All enrolled ALS patients underwent NMUS using a Samsung R10 linear array transducer (LA2-14A, Samsung Medison Co., Ltd., Seoul, South Korea). Muscle thickness measurements were performed as follows:

Tongue (US-TMT): midportion thickness via transoral ultrasound.

Masseter (US-TMM): thickness below mandibular angle, along the muscle long axis.

Biceps brachii (US-BT): mid-upper arm thickness, along the long axis.

First dorsal interosseous (US-TFIDM): dorsal hand measurement, along the muscle long axis.

Rectus femoris (US-RFRMT): resting-state thickness at midpoint between the anterior superior iliac spine and patella superior border.

All measurements were taken with muscles in relaxed state, minimal probe pressure applied, and sites identified via standardized anatomical landmarks. Each measurement was repeated thrice to derive mean values. Limb muscles were assessed bilaterally. For analysis, a single value per feature was retained by selecting the more affected side (e.g., smaller thickness or a more abnormal quantitative measure), to capture the most affected neuromuscular status in each patient.

Concurrently with muscle thickness measurements, grayscale histogram analysis quantified echogenicity in biceps brachii (US-BT), first dorsal interosseous (US-TFIDM), and rectus femoris (US-RFRMT). For each muscle, a region of interest (ROI) was manually selected within the belly (avoiding fascia/bone), and mean grayscale values were extracted. Each measurement was repeated thrice, with final values reported as averages.

All thickness and nerve cross-sectional area (CSA) measurements were obtained on grayscale ultrasound. Echo intensity was quantified using histogram-based mean grayscale values within standardized ROIs.

#### Image acquisition and data curation

All ultrasound examinations were performed by four physicians specialized in ultrasonography, two of whom had 5 years of experience and two with 20 years of experience. M.Z., the most senior physician with 20 years of experience, was responsible for quality control of the images. The operators were blinded to the patients’ clinical evaluations. Each patient underwent MSUS imaging, which was captured and recorded in digital format. Detailed records were kept for each measurement, including muscle thickness (recorded to two decimal places in cm) and histogram ultrasound intensity (measured in grayscale values). To ensure measurement consistency, inter-operator reliability of ultrasound measurements was assessed in a subset of 30 participants using intraclass correlation coefficients. Measurement error was additionally summarized by the standard error of measurement (SEM) and the minimal detectable change at the 95% confidence level (MDC95).

### Data preprocessing

Prior to clustering, we conducted a structured missing-data assessment for all candidate NMUS variables. NMUS features with a missingness rate > 10% were excluded to minimize potential bias and instability in downstream unsupervised analyses. Two ultrasound-derived features were removed at this stage: rectus femoris thickness measured after leg lifting and its CSA.

For the remaining NMUS variables, which each had < 10% missingness, missing values were imputed using multiple imputation by chained equations with the R package mice (predictive mean matching; *m* = 5 imputations) [29]. The completed dataset was subsequently used for clustering and downstream analyses.

For quality control, ultrasound measurements were screened via range checks and distributional inspection; values outside plausible anatomical/technical ranges were flagged and re-checked against source records when available. Because K-means is sensitive to feature scaling, all clustering variables were standardized using z-scores (mean-centered and scaled to unit variance) prior to model fitting. Transformations were not routinely applied unless necessary to reduce extreme skewness; when used, transformations were applied before z-scoring.

### Unsupervised clustering and visualization

Unsupervised phenotyping was performed using K-means clustering on the standardized NMUS feature matrix. To reduce sensitivity to random initialization, K-means was run with multiple random starts (e.g., nstart = 25), and the solution with the lowest within-cluster sum of squares was retained. Euclidean distance in the standardized feature space was used as the underlying dissimilarity measure.

**Fig. 1:**
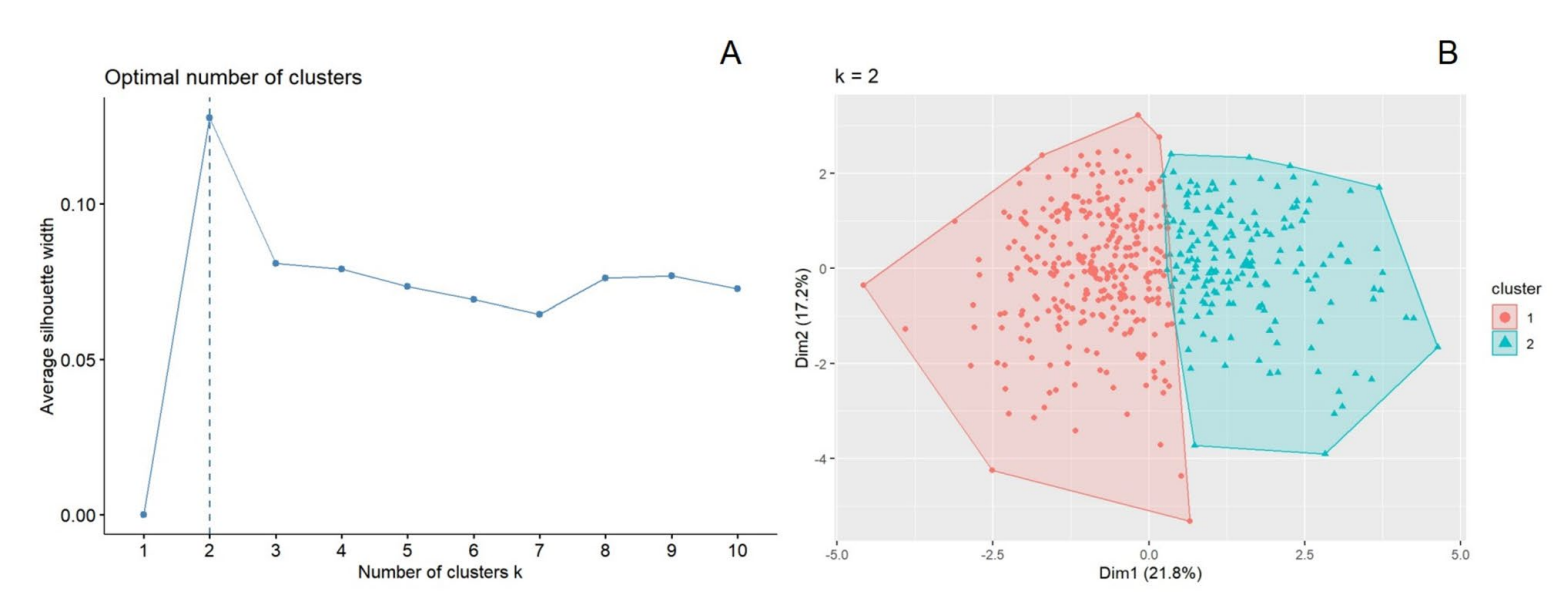
Determination and visualization of ultrasound-based clusters. **A** Plot of average silhouette width versus number of clusters (*k*). The optimal cluster number was determined to be *k* = 2, based on the peak silhouette value, indicating the best-defined structure among tested *k* values. **B** Visualization of K-means clustering results using the first two principal components (Dim1 and Dim2) derived from standardized ultrasound features. Each dot represents a patient, colored by assigned cluster (Mild or Severe)

The optimal number of clusters (*k*) was selected by maximizing the average silhouette width across candidate values of *k* (Fig. 1A). For visualization, we projected participants onto the first two dimensions derived from principal component analysis (PCA) of the standardized NMUS features (Dim1/Dim2) and colored points by cluster assignment (Fig. 1B). The first two principal components (Dim1 and Dim2) represent orthogonal linear combinations of the original ultrasound variables that capture the largest proportion of total variance in the feature space. PCA was used solely for visualization and did not influence the K-means clustering assignments. We additionally visualized the pairwise distance matrix computed from the standardized NMUS features to illustrate the global structure of the feature space (Figure S1). Sensitivity visualizations under alternative *k* values (*k* = 2–5) are provided in Figure S2.

### Statistical analysis

Between-cluster comparisons: Participant characteristics and ultrasound features were summarized overall and by cluster. Continuous variables were compared using two-sided Wilcoxon rank-sum tests, and categorical variables were compared using *χ*^2^ tests or Fisher’s exact tests as appropriate. Cluster-defining ultrasound features were visualized using box-and-jitter plots with significance annotations.

We evaluated the association between ultrasound-derived clusters and ALSFRS-R using multivariable linear regression. Cluster membership was modeled as an ordinal predictor (Mild = 1, Severe = 2; alternatively as a binary indicator in sensitivity analyses), and covariates were prespecified based on clinical relevance, including disease duration (months), sex, age, BMI, hypertension, diabetes, smoking, drinking, speech function, site of onset, and peripheral blood markers (PLT, NEU, MONO, LYM, and ESR). Regression coefficients are reported with 95% confidence intervals and two-sided P values.

Correlation with MMT and EMG features. Spearman rank correlations were used to quantify associations between cluster membership and MMT and EMG features. Correlation coefficients (*ρ*) were displayed as heatmaps with significance markers.

Resampling-based stability. Cluster robustness was assessed via repeated subsampling (e.g., 70–90% of participants per iteration), refitting K-means, and comparing resampled assignments with the reference *k* = 2 solution. Stability was quantified using the mean Jaccard similarity and the adjusted Rand index (ARI).

All tests were two-sided, and statistical significance was defined as *P* < 0.05. Analyses were performed in R (version 4.4.3; key packages included tidyverse, mice, cluster, and factoextra).

## Results

### Participant characteristics

A total of 454 patients with ALS were included, of whom 288 (63.4%) were assigned to the Mild cluster and 166 (36.6%) to the Severe cluster. Overall, 298 patients (65.6%) were male. Compared with the Mild cluster, patients in the Severe cluster were older (54.99 ± 9.23 vs 51.15 ± 9.86 years; *P* < 0.001), had longer disease duration (35.19 ± 23.99 vs 25.53 ± 17.13 months; *P* < 0.001), lower ALSFRS-R scores (22.80 ± 8.90 vs 29.51 ± 8.41; *P* < 0.001), and lower BMI (22.01 ± 3.48 vs 23.85 ± 3.82; *P* < 0.001). Speech function and site of onset did not differ significantly between clusters (*P* = 0.071 and *P* = 0.058, respectively).

For laboratory markers, platelet count, monocyte count, and lymphocyte count were comparable between groups (all *P* > 0.05). Neutrophil count was modestly higher in the Mild cluster than in the Severe cluster (4.27 ± 1.94 vs 3.84 ± 1.31; *P* = 0.011), and ESR was also higher in the Mild cluster (7.24 ± 10.75 vs 5.45 ± 6.82; *P* = 0.014). Smoking, alcohol consumption, and diabetes status did not differ between clusters. Hypertension was more prevalent in the Severe cluster (Table 1).

**Table 1:** Demographic profiles and clinical characteristics in patients with ALS

Characteristic *n* (%) or mean (SD)	All (*n* = 454)	Mild (Cluster1) *n* = 288 weighted %: 63.4	Severe (Cluster 2) *n* = 166 weighted %: 36.6	*P* value
Sex				
Male	298 (65.6)	200 (69.4)	98 (59.0)	0.031*
Female	156 (34.4)	88 (30.6)	68 (41.0)	
Age	52.56 (9.80)	51.15 (9.86)	54.99 (9.23)	< 0.001*
Disease duration (months)	29.06 (20.43)	25.53 (17.13)	35.19 (23.99)	< 0.001*
ALSFRS	27.06 (9.18)	29.51 (8.41)	22.80 (8.90)	< 0.001*
Speech function				
Clear	174 (38.3)	120 (41.7)	54 (32.5)	0.071
Unclear	248 (54.6)	152 (52.8)	96 (57.8)	
Cannot speak	32 (7.0)	16 (5.6)	16 (9.6)	
Site of onset				
Upper limb	237 (52.2)	140 (48.6)	97 (58.4)	0.058
Lower limb	127 (28.0)	84 (29.2)	43 (25.9)	
Upper limb and lower limb	77 (17.0)	56 (19.4)	21 (12.7)	
Bulbar palsy	11 (2.4)	8 (2.8)	3 (1.8)	
Others	2 (0.4)	0 (0.0)	2 (1.2)	
BMI	23.18 (3.80)	23.85 (3.82)	22.01 (3.48)	< 0.001*
PLT	219.34 (55.27)	220.32 (58.09)	218.75 (53.59)	0.487
NEU	4.00 (1.59)	4.27 (1.94)	3.84 (1.31)	0.011*
MONO	0.38 (0.13)	0.37 (0.12)	0.39 (0.15)	0.336
LYM	1.76 (0.56)	1.75 (0.52)	1.77 (0.61)	0.907
ESR	6.12 (8.54)	7.24 (10.75)	5.45 (6.82)	0.014*
Smoking				
No	397 (87.4)	248 (86.1)	149 (89.8)	0.325
Yes	57 (12.6)	40 (13.9)	17 (10.2)	
Drinking				
No	424 (93.4)	269 (93.4)	155 (93.4)	1
Yes	30 (6.6)	19 (6.6)	11 (6.6)	
Hypertension				
No	343 (75.6)	269 (93.4)	108 (65.1)	< 0.001*
Yes	111 (24.4)	19 (6.6)	58 (34.9)	
Diabetes				
No	420 (92.5)	269 (93.4)	151 (91.0)	0.443
Yes	34 (7.5)	19 (6.6)	15 (9.0)	

*PLT* platelet count, *NEU* neutrophil count, *MONO* monocyte count, *LYM* lymphocyte count, *ESR* erythrocyte sedimentation rate

### Ultrasound-derived clusters and determination of *k*

To identify data-driven phenotypic subgroups, we performed K-means clustering using the pre-specified NMUS feature set. The optimal number of clusters was evaluated across candidate *k* values using the average silhouette width. As shown in Fig. 1A, the silhouette profile reached its maximum at *k* = 2, indicating the best-defined clustering structure among the tested solutions; therefore, the two-cluster model was selected for subsequent analyses.

The resulting two-cluster solution is visualized in Fig. 1B using a two-dimensional projection based on the first two principal components derived from the NMUS feature space. Each point represents an individual patient, colored by cluster assignment, with shaded polygons indicating the approximate cluster boundaries. The two clusters showed clear separation predominantly along Dim1, supporting the presence of distinct ultrasound-derived phenotypes. Visual comparisons under alternative *k* settings (*k* = 3–5) are provided in Figure S2, showing increased overlap and less coherent separation relative to the chosen *k* = 2 solution.

### Distinct neuromuscular ultrasound profiles between clusters

**Fig. 2:**
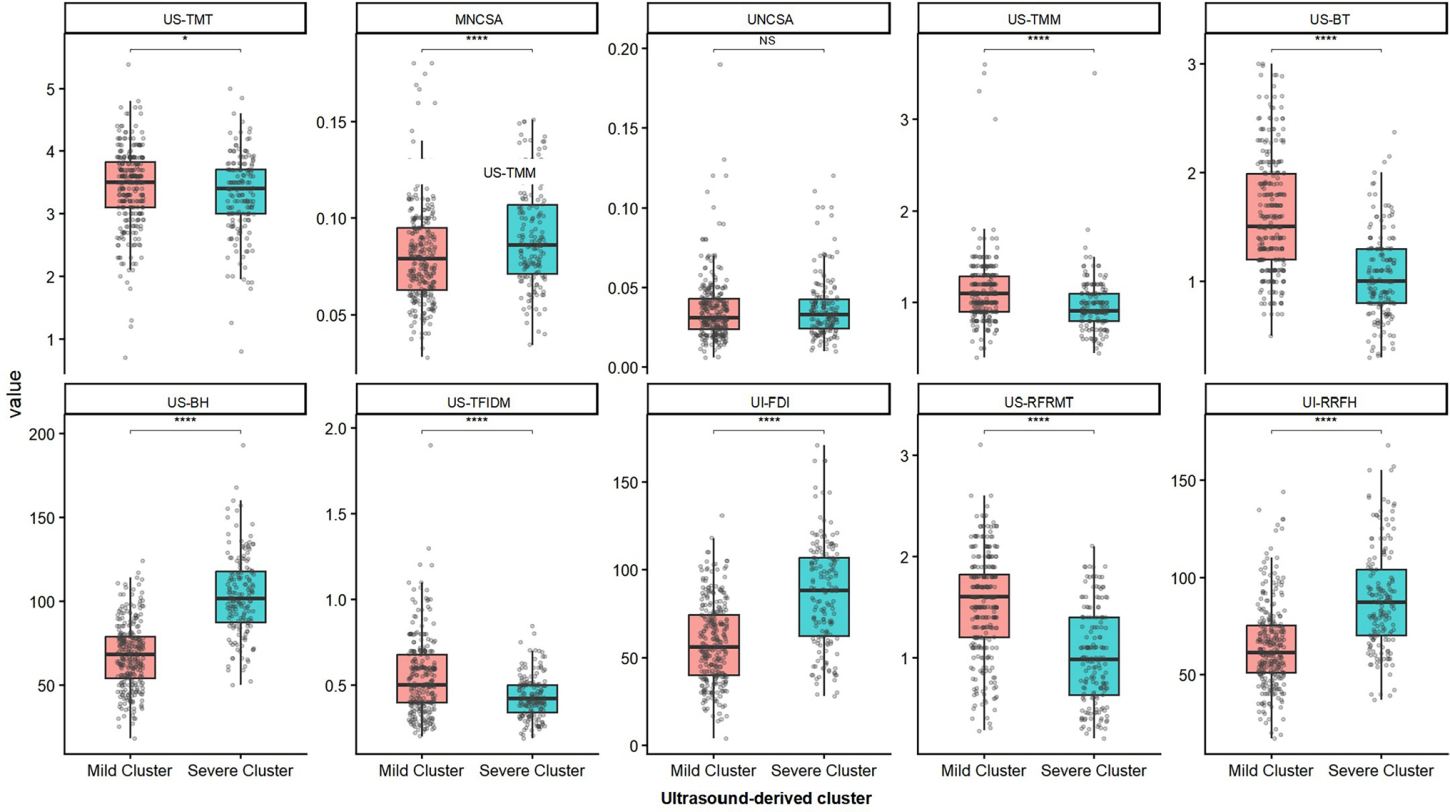
Differences in neuromuscular ultrasound features between ultrasound-derived clusters. Faceted box-and-jitter plots compare ten neuromuscular ultrasound (NMUS) features between the two ultrasound-derived clusters (Mild and Severe). Each dot represents an individual patient. Boxes indicate the interquartile range (IQR) with the median shown as the center line; whiskers extend to 1.5 × IQR. Between-cluster differences for each feature were evaluated using two-sided Wilcoxon rank-sum tests. Statistical significance is annotated as *P* < 0.05 (), *P* < 0.01 (), *P* < 0.001 (), and *P* < 0.0001 (****); NS indicates not significant. *US-TMT* tongue muscle thickness, *US-TMM* masseter muscle thickness, *US-BT* biceps brachii thickness, *UI-BH* biceps brachii echo intensity (histogram-based mean grayscale value), *US-TFIDM* first dorsal interosseous thickness, *UI-FDI* first dorsal interosseous echo intensity (histogram-based mean grayscale value), *US-RFRMT* rectus femoris thickness at rest, *UI-RRFH* rectus femoris echo intensity at rest (histogram-based mean grayscale value); MNCSA/UNCSA, median/ulnar nerve cross-sectional area

Among the ultrasound-derived subgroups, the Mild and Severe clusters showed distinct distributions across the cluster-defining NMUS features (Fig. 2; Table S1).

Compared with the Mild cluster, the Severe cluster showed significantly different distributions in most cluster-defining ultrasound metrics, including tongue thickness (US-TMT; *P* = 0.036), masseter thickness (US-TMM; *P* < 0.001), biceps thickness (US-BT; *P* < 0.001), and first dorsal interosseous thickness (US-TFIDM; *P* < 0.001).

Echogenicity/echo-intensity indices also differed between clusters, including biceps (UI-BH; *P* < 0.001) and FDI (UI-FDI; *P* < 0.001). Rectus femoris measurements (US-RFRMT and UI-RRFH) and median nerve cross-sectional area (MNCSA) were likewise significantly different across clusters (all *P* < 0.001), whereas ulnar nerve CSA (UNCSA) did not differ (*P* = 0.588). Full distributions are summarized in Table S1 and visualized in Fig. 2.

### Association between cluster membership and functional status (ALSFRS-R)

In linear regression models with ALSFRS-R as the dependent variable, ultrasound-derived cluster membership was significantly associated with functional status. In the crude model, patients in the Severe cluster had substantially lower ALSFRS-R scores than those in the Mild cluster (*β* = − 6.718, 95% CI − 8.364 to − 5.072; *P* < 0.001). The association between cluster membership and ALSFRS-R remained significant after adjustment for disease duration, demographics, vascular/metabolic comorbidities, lifestyle factors, speech function, site of onset, and inflammatory markers (adjusted *β* = − 3.841, 95% CI − 5.410 to − 2.273; *P* < 0.001), indicating that the ultrasound-derived phenotype was independently related to worse functional status(Table 2).

**Table 2:** Multivariable linear regression of ALSFRS-R by ultrasound-derived cluster

	Crude model		Adjusted model	
	Crude OR (95% CI)	*P*-value	Adjusted OR (95% CI)	*P*-value
Cluster				
Mild cluster	Ref		Ref	
Severe cluster	−6.718 (−8.364, −5.072)	< 0.001***	−3.841 (−5.410, −2.273)	< 0.001***
Duration (month)	−0.086 (−0.127, −0.045)	< 0.001***	−0.058 (−0.092, −0.024)	< 0.001***
Sex				
Male	Ref		Ref	
Female	−0.986 (−2.767, 0.796)	0.278	−0.067 (−1.663, 1.529)	0.934
Age	−0.088 (−0.174, −0.002)	0.045*	0.017 (−0.058, 0.092)	0.658
BMI	0.432 (0.212, 0.651)	< 0.001***	0.311 (0.088, 0.535)	0.014*
Hypertension				
No	Ref		Ref	
Yes	−3.236 (−5.184, −1.287)	0.001***	−2.187 (−3.852, −0.522)	0.010*
Diabetes				
No	Ref		Ref	
Yes	−0.889 (−4.106, 2.329)	0.588	−1.271 (−3.891, 1.350)	0.341
Smoking				
No	Ref		Ref	
Yes	1.961 (−0.590, 4.512)	0.132	0.073 (−2.385, 2.531)	0.953
Drinking				
No	Ref		Ref	
Yes	1.509 (−1.899, 4.917)	0.385	0.071 (−3.161, 3.303)	0.966
Speech function				
Clear	Ref		Ref	
Unclear	−6.872 (−8.422, −5.323)	< 0.001***	−6.652 (8.093, −5.212)	< 0.001***
Cannot speak	−16.540 (−19.553, −13.526)	< 0.001***	−14.777 (−17.726, −11.828)	< 0.001***
Site of onset				
Upper limb	Ref		Ref	
Lower limb	−0.853 (−2.834, 1.129)	0.398	−1.313 (−2.888, 0.263)	0.102
Upper limb and lower limb	−0.402 (−2.766, 1.961)	0.738	2.410 (0.418, 4.401)	0.018*
Bulbar palsy	−4.052 (−9.610, 1.506)	0.153	−2.018 (−6.455, 2.418)	0.372
Others	−10.006 (−22.802, 2.789)	0.125	−5.029 (−15.152, 5.093)	0.329
PLT	−0.004 (−0.019, 0.012)	0.646	0.005 (−0.009, 0.019)	0.465
NEU	−1.325 (−1.844, −0.805)	< 0.001***	−0.357 (−0.961, 0.248)	0.247
MONO	−11.513 (−17.923, −5.104)	< 0.001***	−9.082 (−16.112, −2.052)	0.011*
LYM	1.324 (−0.199, 2.847)	0.088	1.537 (0.203, 2.871)	0.024
ESR	−0.230 (−0.328, −0.132)	< 0.001***	−0.085 (−0.178, 0.008)	0.074

Note: Crude model included cluster membership only. Adjusted model additionally adjusted for duration (months), sex, age, BMI, hypertension, diabetes, smoking, drinking, speech function, site of onset, platelet count (PLT), neutrophil count (NEU), monocyte count (MONO), lymphocyte count (LYM), and erythrocyte sedimentation rate (ESR)

Among covariates in the adjusted model, longer disease duration was associated with lower ALSFRS-R (*β* per month = − 0.058, 95% CI − 0.092 to − 0.024; *P* < 0.001), whereas BMI was positively associated with ALSFRS-R (*β* = 0.311, 95% CI 0.088 to 0.535; *P* = 0.014). Hypertension was associated with lower ALSFRS-R (*β* = − 2.187, 95% CI − 3.852 to − 0.522; *P* = 0.010). Compared with clear speech, unclear speech (*β* = − 6.652, 95% CI − 8.093 to − 5.212; *P* < 0.001) and inability to speak (*β* = − 14.777, 95% CI − 17.726 to − 11.828; *P* < 0.001) were strongly associated with lower ALSFRS-R. Regarding laboratory markers, MONO remained negatively associated with ALSFRS-R (*β* = − 9.082, 95% CI − 16.112 to − 2.052; *P* = 0.011), while LYM showed a positive association (*β* = 1.537, 95% CI 0.203 to 2.871; *P* = 0.024); NEU and ESR were not significant after adjustment.

### Correlations of clusters with muscle strength and electromyographic features

**Fig. 3:**
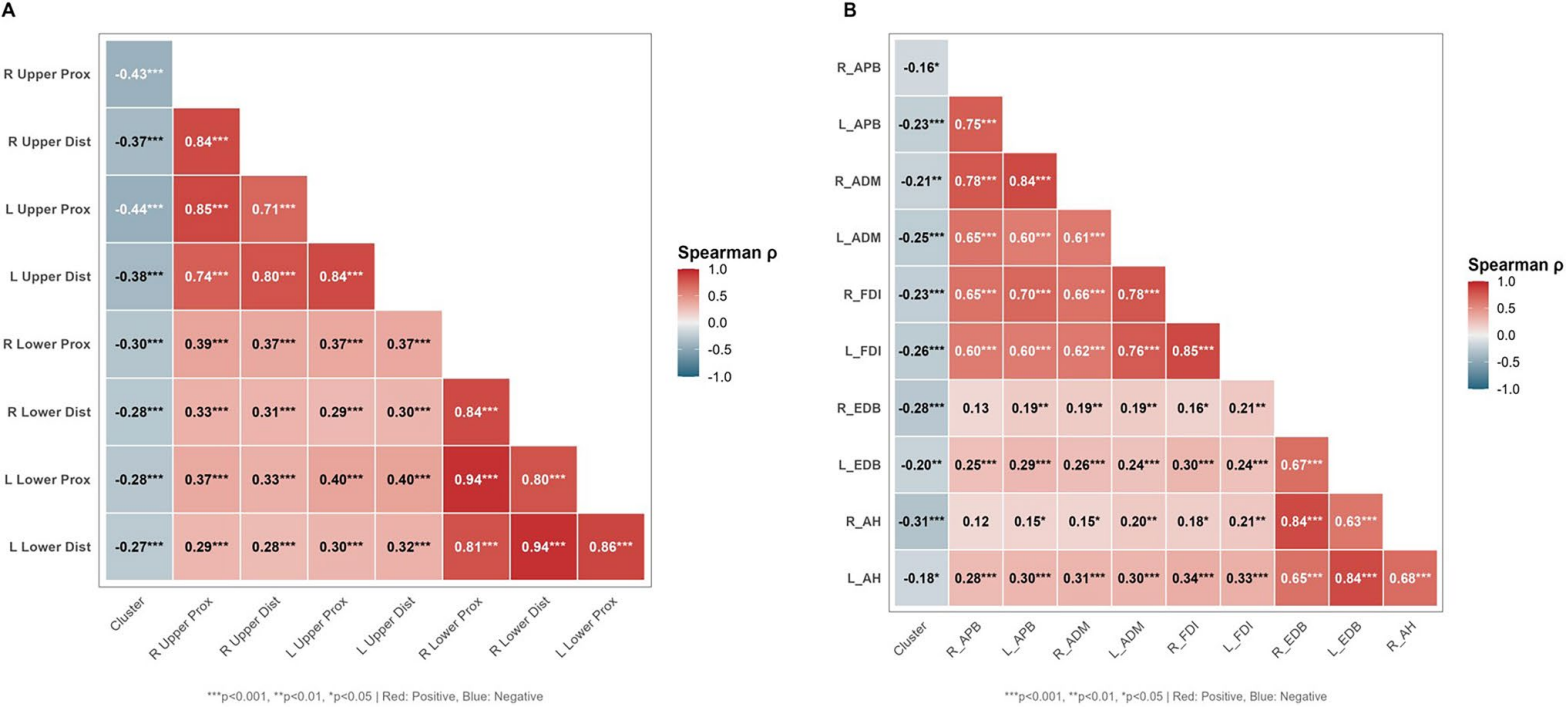
Correlations of ultrasound-derived clusters with muscle strength and electromyographic features. Heatmaps show Spearman rank correlations (*ρ*) between ultrasound-derived cluster membership and **A** manual muscle testing (MMT) features and **B** electromyographic (EMG) features. Cluster membership was coded as an ordinal variable (Mild = 1, Severe = 2). Cells display correlation coefficients, and the color scale indicates the direction and magnitude of ρ (red, positive; blue, negative). Statistical significance is denoted as *P < 0.05, **P < 0.01, and ***P < 0.001 (two-sided)

Ultrasound-derived cluster membership (coded as Mild = 1, Severe = 2) showed consistent associations with functional impairment on both clinical strength testing and electrophysiological measures (Fig. 3; Table S2). For MMT, cluster membership was inversely correlated with proximal and distal muscle strength in the upper and lower limbs (Spearman *ρ* ranging from − 0.27 to − 0.44; all *P* < 0.001; Fig. 3A), indicating broadly lower MMT scores in the Severe cluster. Similarly, cluster membership was negatively correlated with CMAP amplitudes across multiple examined muscles/nerves, including APB, ADM, FDI, EDB, and AH (*ρ* = − 0.16 to − 0.31; *P* < 0.05 to *P* < 0.001; Fig. 3B), supporting that the Severe cluster captured a phenotype with more pronounced lower motor neuron dysfunction.

### Sensitivity and stability of the two-cluster solution

To evaluate whether the identified structure depended on the choice of *k*, we visualized alternative K-means solutions (*k* = 2–5) in the same two-dimensional projection space (Figure S2). The *k* = 2 solution showed the clearest global separation with minimal overlap, whereas increasing *k* primarily subdivided the original groups into smaller partitions with greater boundary overlap and less distinct separation, suggesting no additional stable higher-order structure beyond two clusters.

We further assessed the robustness of the *k* = 2 clustering using resampling-based stability analysis (Figure S3). Across repeated resampling runs, concordance between the resampled cluster assignments and the original reference solution remained consistently high, with the mean Jaccard similarity (averaged across the two clusters) concentrated toward the upper range and the adjusted Rand index (ARI) similarly skewed toward high values. Together, these results indicate that the two-cluster solution is not an artifact of sampling variability but reflects a reproducible partition of the NMUS feature space.

### Exploratory correlation structure across clinical/inflammatory/ultrasound features

To explore the broader interrelationships among ultrasound-derived clustering features, clinical characteristics, and peripheral inflammatory markers, we conducted a pairwise Spearman correlation analysis (Figure S4).

Cluster-defining ultrasound features showed modest but significant correlations with several clinical and biological variables. Specifically, they were negatively correlated with ALSFRS-R scores (*ρ* = − 0.35, *P* < 0.001) and positively associated with age (*ρ* = 0.19, *P* < 0.001) and hypertension (*ρ* = 0.11, *P* = 0.018), suggesting that patients in the more severe ultrasound-defined phenotype tended to be older and functionally more impaired.

Among inflammatory indices, NEU (*ρ* = 0.57, *P* < 0.001) and MONO (*ρ* = 0.35, *P* < 0.001) were positively correlated, while ESR showed an inverse relationship with ALSFRS-R (*ρ* = − 0.24, *P* < 0.001), reflecting that systemic inflammation was linked to worse functional performance. Additionally, speech function displayed a strong positive association with ALSFRS-R (*ρ* = 0.48, *P* < 0.001), further validating the consistency between the clinical and imaging-based disease severity markers.

Overall, this exploratory correlation matrix demonstrates coherent relationships among functional, inflammatory, and ultrasound-based parameters, reinforcing the biological plausibility of the clustering-derived ALS subtypes.

In addition, quality control analyses confirmed the reproducibility of the ultrasound measurements. Inter-operator reliability was high, with ICC values ranging from 0.878 to 0.947, including good reliability for median and ulnar nerve CSA (ICC 0.878–0.880). Detailed reliability metrics are provided in Supplementary Table S3.

## Discussion

In this large real-world cohort of 454 patients with ALS, we show that an ultrasound-driven, unsupervised clustering approach can identify clinically meaningful phenotypic subgroups using a pre-specified panel of NMUS features. The optimal number of clusters was supported by silhouette optimization (Fig. 1A) and clear separation in a two-dimensional projection of the feature space (Fig. 1B), yielding a Mild (Cluster 1) and Severe (Cluster 2) subgroup (Table S1). Importantly, these ultrasound-defined clusters were not merely descriptive: cluster membership was independently associated with functional status as measured by ALSFRS-R after adjustment for demographic and clinical covariates (Table 2), and showed coherent relationships with MMT and electrophysiologic measures (Fig. 3; Table S2). The overall multivariate structure of the clustering feature space also suggested non-random subgrouping (Fig. S1), and resampling-based analyses demonstrated high stability of the two-cluster solution (Fig. S3), with sensitivity analyses across alternative *k* values providing complementary support (Fig. S2). Collectively, our results indicate that NMUS-based clustering could serve as a practical framework to help quantify ALS phenotypic heterogeneity beyond conventional clinical descriptors.

A key observation is that the two subgroups differed by a consistent pattern of muscle and nerve ultrasound alterations, rather than by a single isolated marker. Across the clustering feature set, the Severe cluster tended to exhibit thinner muscles (e.g., tongue, biceps brachii, first dorsal interosseous, and rectus femoris thickness measures) alongside higher grayscale echo-intensity metrics in limb muscles (Fig. 2; Table S1). This joint pattern is biologically plausible: reductions in muscle thickness are compatible with atrophy, whereas increased echo intensity on B-mode ultrasound is commonly interpreted as reflecting architectural remodeling with greater fatty and fibrotic replacement of contractile tissue. In parallel, median nerve cross-sectional area differed between clusters, whereas ulnar nerve cross-sectional area showed weaker or non-significant separation in the present dataset (Fig. 2; Table S1). Consistent with prior work, nerve ultrasound has been proposed as a non-invasive modality to characterize peripheral nerve involvement and potentially monitor disease burden in ALS [30], and our findings further support the concept of substantial phenotypic heterogeneity in ALS [31, 32]. From a pathophysiological perspective, progressive motor neuron degeneration leads to denervation and downstream muscle wasting, which may manifest on ultrasound as reduced muscle thickness and increased echogenicity [33]. MNCSA was slightly higher in the Severe cluster. Because median nerve CSA at the wrist is sensitive to entrapment-related swelling (e.g., carpal tunnel syndrome) and can be influenced by patient-level factors such as BMI or diabetes, this between-cluster difference should be interpreted cautiously and verified in future studies incorporating CTS (carpal tunnel syndrome) screening and standardized measurement landmarks [34]. Taken together, integrating multi-site muscle thickness, quantitative echogenicity, and nerve CSA provides a practical way to capture clinically relevant phenotypic heterogeneity in ALS at a single time point, consistent with prior work supporting nerve and muscle ultrasound as non-invasive tools to characterize peripheral neuromuscular burden in ALS.

The clinical relevance of this ultrasound-defined stratification is supported by its consistent association with functional status. In multivariable linear regression, ultrasound-derived cluster membership remained independently associated with ALSFRS-R after adjustment for demographic factors, disease-related variables, comorbidities, and laboratory markers (adjusted *β* ≈ − 3.8 points for the Severe vs. Mild cluster, *P* < 0.001; Table 2). This finding is in line with the broader literature suggesting that neuromuscular ultrasound can capture clinically meaningful disease burden in ALS and may complement conventional clinical assessments [35]. Several covariates also showed expected associations with ALSFRS-R, including longer disease duration and worse speech function (Table 2), supporting the internal coherence of the model. Although causality cannot be inferred from this retrospective cross-sectional design, the persistence of the cluster–ALSFRS-R association in the fully adjusted model suggests that ultrasound-derived phenotyping provides incremental information not fully reflected by conventional clinical variables alone.

From a methodological standpoint, our approach also aligns with prior efforts to address ALS heterogeneity using unsupervised methods (e.g., latent class/cluster-based phenotyping), although most previous work relied primarily on clinical trajectories or symptom profiles rather than imaging-derived neuromuscular features [36, 37]. More broadly, recent machine-learning initiatives in ALS have increasingly aimed to stratify individual patients into diagnostic, phenotypic, or prognostic categories using multimodal data [38]. Studies relying on brain data alone have highlighted that MRI-driven subtyping can be challenging and may not fully capture peripheral neuromuscular involvement [39]. Against this backdrop, incorporating bedside measures of peripheral neuromuscular structure (e.g., NMUS-derived muscle thickness, echogenicity, and nerve CSA) may provide complementary information to improve patient categorization and clinical interpretability.

Concordant findings emerged when we examined strength and electrophysiologic correlates. Cluster membership (coded ordinally as Mild = 1, Severe = 2) showed consistent negative Spearman correlations with multiple MMT measures across upper and lower limbs, indicating lower muscle strength in the Severe cluster (Fig. 3A; Table S2). Similarly, electrophysiologic features demonstrated coherent associations with the Severe cluster across several nerves/muscles, with the directionality broadly compatible with greater motor unit/axonal involvement in the Severe subgroup (Fig. 3B; Table S2). Prior work supports the biological plausibility of this cross-modal alignment: neuromuscular ultrasound has been increasingly positioned as a complementary bedside tool to electrophysiology in ALS, capturing structural and dynamic muscle changes (e.g., atrophy patterns and fasciculations) that relate to neurogenic involvement [40]. In particular, ultrasound-based detection of fasciculations has been shown to add diagnostic value and improve sensitivity compared with relying on EMG alone in some settings, reinforcing that ultrasound and EMG provide partially non-redundant information [41, 42]. Taken together, the convergence of ultrasound-derived clustering with both bedside strength assessments and EMG/CMAP-derived measures lends supportive (though still observational) evidence that the identified clusters reflect differences in underlying neuromuscular involvement rather than purely statistical artifacts of the ultrasound feature space.

A practical contribution of this work is that it provides an image-derived, clinic-ready stratification approach that may complement traditional ALS classification based on onset site, symptom profile, or clinical staging. Clinical staging systems and functional scales (e.g., Kings/MiToS frameworks derived from ALSFRS-R components) remain central for prognosis and trial endpoints, but they primarily reflect clinical burden and functional involvement and do not directly quantify peripheral tissue architecture [43]. By contrast, NMUS offers a non-invasive window into muscle and nerve structure, is repeatable at the bedside, and can provide quantitative readouts (e.g., thickness, CSA, and histogram-based echo intensity) that are relevant to neuromuscular involvement [44]. Beyond phenotypic stratification, NMUS may also support differential diagnosis in early motor neuron disorders. For example, ALS can clinically overlap with spinal and bulbar muscular atrophy (SBMA, Kennedy’s disease) before androgen receptor CAG repeat expansions are confirmed [45]. Similarly, distinguishing primary lateral sclerosis (PLS) from ALS at first review may be challenging, yet lower motor neuron involvement is typically absent or minimal in PLS [46]. Therefore, the presence (or relative absence) of NMUS abnormalities consistent with neurogenic muscle change may provide additional objective support when differentiating these conditions in early-stage presentations. Quantitative ultrasound signatures of neurogenic muscle remodeling (e.g., reduced thickness with increased echogenicity across multiple sites) may provide supportive bedside evidence that complements clinical assessment and electrophysiology. Furthermore, ALS-specific clinical patterns such as split-hand and split-limb phenomena are often considered early diagnostic cues [47] and have been linked to selective vulnerability shaped by corticospinal innervation [48]. While our study did not directly quantify split-hand metrics, NMUS-derived peripheral neuromuscular signatures may provide a complementary, tissue-level perspective on selective involvement across motor units. In this context, our findings suggest that a compact NMUS panel can be leveraged to derive ultrasound-informed subgroups that track with functional impairment (Table 2) and neuromuscular performance (Fig. 3; Table S2). This type of stratification may be useful for risk communication, for enriching clinical trials with participants more likely to show measurable progression, or for tailoring follow-up intensity—hypotheses that warrant prospective evaluation.

A strength of the study is the attention to robustness and validation, which is often a limiting aspect of unsupervised phenotyping studies. The two-cluster solution was supported by silhouette-based selection (Fig. 1A) and by visualization of clustering structure in a low-dimensional projection (Fig. 1B). Such silhouette criteria and dimensionality reduction approaches have been increasingly recommended in clustering studies to avoid overfitting and enhance interpretability. To contextualize the multivariate structure, we additionally provide a pairwise distance matrix heatmap that illustrates block-like similarity patterns in the standardized clustering feature space (Fig. S1). We further explored sensitivity to alternative *k* values (*k* = 2–5), showing how apparent separation and overlap change across candidate solutions (Fig. S2). Most importantly, we performed resampling-based stability assessments: both the mean Jaccard similarity and adjusted Rand index (ARI) distributions were high across repeated subsampling runs (Fig. S3), supporting that the *k* = 2 partition is stable under moderate perturbations of the sample. Resampling or bootstrap stability measures have been advocated as key validation steps for unsupervised phenotype discovery to reduce the risk of sampling bias [49].

We also examined the broader correlation structure across clinical variables, inflammatory markers, and clustering-related features as an exploratory analysis (Fig. S4). The correlation matrix suggests that the ultrasound-derived clustering signal is related to functional status and disease duration, while showing only modest correlations with several demographic factors and laboratory inflammatory indices (Fig. S4). Similar exploratory correlation approaches have been used in other ALS biomarker studies to map how imaging or laboratory features co-vary with clinical measures [50]. This pattern is consistent with the notion that NMUS features may capture a component of disease burden that is not fully reducible to standard clinical variables. However, we emphasize that these associations are exploratory and should not be interpreted as evidence of mechanistic pathways; rather, they provide a descriptive overview of how measured domains co-vary in this cohort and may help generate hypotheses for longitudinal and multi-omic studies, where causal inference and temporal dynamics can be more rigorously examined.

Several limitations should be acknowledged. First, the retrospective and cross-sectional design precludes inference about temporal trajectories and prognostic value. Whether NMUS-derived clusters predict subsequent progression rate, survival, or treatment response requires prospective follow-up. Second, this was a single-center study, and external validation in independent cohorts and across devices/operators will be important for generalizability. Third, although we adopted a transparent missing-data strategy—excluding ultrasound features with > 10% missingness and using multiple imputation for the remaining features with < 10% missingness—imputation may still introduce uncertainty, particularly if missingness is not completely at random. Clinical management variables (PEG, non-invasive ventilation, and riluzole use) were incompletely documented and were not analyzed; future prospective studies should capture them systematically. Laterality was not explicitly modeled, and future prospective studies should systematically evaluate side-to-side differences and their clinical implications. Finally, clustering results can be influenced by feature scaling and selection; while we used a pre-specified NMUS feature set and conducted stability and sensitivity checks, future work should explicitly test the reproducibility of clustering under alternative preprocessing pipelines and feature subsets.

## Conclusion

Two different ALS subtypes were identified in this study, based on NMUS parameters and using unsupervised clustering analysis, which provided new insights into ALS disease heterogeneity. By integrating NMUS features into an unsupervised clustering framework, we provide preliminary evidence that imaging-derived neuromuscular profiles can help characterize ALS heterogeneity beyond traditional approaches that rely primarily on clinical phenotypes or genotyping. This approach offers a novel perspective for uncovering the heterogeneity of ALS and lays the groundwork for future integrative analysis of multimodal data. More complex models that incorporate genetic, metabolic, and imaging data may further refine the subtyping of ALS and advance the field of precision medicine. Future studies are warranted to validate and extend these observations using larger cohorts and integration of multimodal data, including genetic and metabolic information, to refine patient subtyping and facilitate the development of precision medicine in ALS.

## Supplementary Information

Below is the link to the electronic supplementary material.

Supplementary file1 (DOCX 14 KB)

Supplementary file2 (DOCX 24 KB)

Supplementary file3Figure S1. Pairwise distance matrix heatmap of the clustering feature space. A heatmap of the pairwise distance matrix computed from the standardized clustering feature set (NMUS variables). Each row/column represents one participant, and each cell indicates the distance between a pair of participants (cool colors, smaller distances/more similar profiles; warm colors, larger distances/more dissimilar profiles). The diagonal corresponds to self-distance (zero). Apparent block-like patterns reflect subgroups of participants with relatively similar multivariate ultrasound profiles. This visualization is provided to illustrate the overall structure of the feature space and was not used as a criterion to determine cluster number (JPEG 7672 KB)

Supplementary file4Figure S2. Cluster separation across different numbers of clusters (k = 2–5). Two-dimensional projection of participants based on the NMUS clustering features, visualized under different k values (k = 2, 3, 4, and 5). Points represent individual participants and are colored by their assigned cluster at each k. Shaded polygons indicate the approximate convex hull (cluster boundary) of each cluster in the projected space. The displayed axes (Dim1 and Dim2) correspond to the first two dimensions of the visualization space (e.g., principal components or a similar linear projection used for display), and the percentage values indicate the proportion of variance explained by each dimension. This figure is provided as a supplementary visualization to compare the apparent separation and overlap of clusters under alternative k settings, whereas the primary selection of k in the main text was based on the pre-specified criterion (e.g., silhouette optimization) (PNG 119 KB)

Supplementary file5Figure S3. Resampling-based stability of the ultrasound-derived two-cluster solution. (A) Histogram of the mean Jaccard similarity (averaged across the two clusters) comparing cluster membership from each resampling run with the original k = 2 reference clustering. (B) Histogram of the adjusted Rand index (ARI) comparing resampling-based cluster assignments with the original k = 2 solution (JPG 89 KB)

Supplementary file6Figure S4. Spearman correlation matrix of clinical characteristics, inflammatory markers, and cluster-defining features. Spearman correlation matrix across clinical characteristics (e.g., demographics and disease-related variables), inflammatory markers, and cluster-defining features. Colors represent Spearman’s ρ (red, positive; blue, negative). The diagonal indicates self-correlations. Significance is annotated as *P < 0.05, **P < 0.01, and ***P < 0.001 (two-sided) (JPEG 1308 KB)

Supplementary file7 (DOCX 14 KB)

## Data Availability

The datasets generated and/or analyzed during this study are available from the corresponding author upon reasonable request.
