# Supplementary material for: Imaging-derived neuromuscular ultrasound phenotypes are associated with functional status in amyotrophic lateral sclerosis
Source: J Neurol. 2026 Feb 21;273(2):158. doi: 10.1007/s00415-026-13705-4 (PMC12924791; doi:10.1007/s00415-026-13705-4)
Supplement: Supplementary file 1 — Supplementary file1 (DOCX 14 KB) [file 415_2026_13705_MOESM1_ESM.docx]

Supplementary Table S1. Ultrasound features used for clustering and their distributions across ultrasound-derived clusters

| Characteristic | All (n = 454) | Mild  ( Cluster 1) n = 288 Weighted %: 63.4 | Severe  (Cluster 2) n = 166 Weighted %: 36.6 | p value |
| --- | --- | --- | --- | --- |
| n (%) or mean (SD) |  |  |  |  |
| US-TMT | 3.38 (0.63) | 3.43 (0.62) | 3.30 (0.64) | 0.036 |
| US-TMM | 1.07 (0.35) | 1.12 (0.36) | 0.98 (0.30) | <0.001 |
| US-BT | 1.40 (0.56) | 1.61 (0.53) | 1.04 (0.39) | <0.001 |
| UI-BH | 80.70 (27.37) | 78.65 (28.85) | 103.55 (24.02) | <0.001 |
| US-TFIDM | 0.51 (0.20) | 0.55 (0.22) | 0.43 (0.12) | <0.001 |
| UI-FDI | 68.53 (29.06) | 58.28 (23.01) | 86.30 (29.95) | <0.001 |
| US-RFRMT | 1.34 (0.55) | 1.53 (0.50) | 1.01 (0.47) | <0.001 |
| UI-RRFH | 73.30 (26.19) | 64.38 (21.51) | 88.77 (26.45) | <0.001 |
| MNCSA | 0.08 (0.03) | 0.08 (0.03) | 0.09 (0.03) | <0.001 |
| UNCSA | 0.04 (0.02) | 0.04 (0.02) | 0.04 (0.02) | 0.588 |

Data are presented as mean (SD) unless otherwise indicated. Ultrasound-derived clusters were obtained using unsupervised clustering based on the neuromuscular ultrasound (NMUS) feature set listed in this table. Continuous variables were compared between clusters using two-sided statistical tests as specified in the Methods; P values < 0.05 were considered statistically significant. “Weighted %” indicates the proportion of participants in each cluster after applying the analytic weights (if applicable). Abbreviations: US-TMT, tongue muscle thickness; US-TMM, masseter muscle thickness; US-BT, biceps brachii thickness; UI-BH, biceps brachii echogenicity/echo intensity (histogram-based index); US-TFIDM, first dorsal interosseous muscle thickness; UI-FDI, first dorsal interosseous echogenicity/echo intensity; US-RFRMT, rectus femoris muscle thickness; UI-RRFH, rectus femoris echogenicity/echo intensity; MNCSA, median nerve cross-sectional area; UNCSA, ulnar nerve cross-sectional area.
