# Supplementary material for: Imaging-derived neuromuscular ultrasound phenotypes are associated with functional status in amyotrophic lateral sclerosis
Source: J Neurol. 2026 Feb 21;273(2):158. doi: 10.1007/s00415-026-13705-4 (PMC12924791; doi:10.1007/s00415-026-13705-4)
Supplement: Supplementary file 2 — Supplementary file2 (DOCX 24 KB) [file 415_2026_13705_MOESM2_ESM.docx]

Supplementary Table S2. Manual muscle testing and electromyographic features by ultrasound-derived cluster in patients with ALS

| Characteristic | | All (n = 454) | Mild  ( Cluster 1) n = 288 Weighted %: 63.4 | Severe  (Cluster 2) n = 166 Weighted %: 36.6 | p value |
| --- | --- | --- | --- | --- | --- |
| n (%) or mean (SD) | |  |  |  |  |
| Right Upper limb muscle strength grading(proximal ) | Grade 0 | 26 ( 5.7) | 4 ( 1.4) | 22 ( 13.3) | <0.001* |
|  | Grade 1 | 65 ( 14.3) | 21 ( 7.3) | 44 ( 26.5) |  |
|  | Grade 2 | 98 ( 21.6) | 56 ( 19.4) | 42 ( 25.3) |  |
|  | Grade 3 | 94 ( 20.7) | 63 ( 21.9) | 31 ( 18.7) |  |
|  | Grade 4 | 108 ( 23.8) | 86 ( 29.9) | 22 ( 13.3) |  |
|  | Grade 5 | 63 ( 13.9) | 58 ( 20.1) | 5 ( 3.0) |  |
| Right Upper limb muscle strength grading（distal） | Grade 0 | 20 ( 4.4) | 5 ( 1.7) | 15 ( 9.0) | <0.001* |
|  | Grade 1 | 60 (13.2) | 20 ( 6.9) | 40 ( 24.1) |  |
|  | Grade 2 | 95 (20.9) | 52 ( 18.1) | 43 ( 25.9) |  |
|  | Grade 3 | 117 (25.8) | 79 ( 27.4) | 38 ( 22.9) |  |
|  | Grade 4 | 104 (22.9) | 79 ( 27.4) | 25 ( 15.1) |  |
|  | Grade 5 | 58 (12.8) | 53 ( 18.4) | 5 ( 3.0) |  |
| Right Lower limb muscle strength grading(proximal ） | Grade 0 | 19 ( 4.2) | 5 ( 1.7) | 14 ( 8.4) | <0.001* |
|  | Grade 1 | 30 ( 6.6) | 5 ( 1.7) | 25 ( 15.1) |  |
|  | Grade 2 | 59 (13.0) | 36 ( 12.5) | 23 ( 13.9) |  |
|  | Grade 3 | 83 (18.3) | 45 ( 15.6) | 38 ( 22.9) |  |
|  | Grade 4 | 170 (37.4) | 125 ( 43.4) | 45 ( 27.1) |  |
|  | Grade 5 | 93 (20.5) | 72 ( 25.0) | 21 ( 12.7) |  |
| Right Lower limb muscle strength grading（distal） | Grade 0 | 24 ( 5.3) | 9 ( 3.1) | 15 ( 9.0) |  |
|  | Grade 1 | 30 ( 6.6) | 8 ( 2.8) | 22 ( 13.3) |  |
|  | Grade 2 | 56 (12.3) | 30 ( 10.4) | 26 ( 15.7) | <0.001* |
|  | Grade 3 | 88 (19.4) | 52 ( 18.1) | 36 ( 21.7) |  |
|  | Grade 4 | 153 (33.7) | 108 ( 37.5) | 45 ( 27.1) |  |
|  | Grade 5 | 103 (22.7) | 81 ( 28.1) | 22 ( 13.3) |  |
| Left Upper limb muscle strength grading(proximal ) | Grade 0 | 26 ( 5.7) | 6 ( 2.1) | 20 ( 12.0) | <0.001* |
|  | Grade 1 | 60 (13.2) | 19 ( 6.6) | 41 ( 24.7) |  |
|  | Grade 2 | 100 (22.0) | 51 ( 17.7) | 49 ( 29.5) |  |
|  | Grade 3 | 101 (22.2) | 68 ( 23.6) | 33 ( 19.9) |  |
|  | Grade 4 | 108 ( 23.8) | 89 ( 30.9) | 19 ( 11.4) |  |
|  | Grade 5 | 59 (13.0) | 55 ( 19.1) | 4 ( 2.4) |  |
| Left Upper limb muscle strength grading（distal） | Grade 0 | 20 ( 4.4) | 5 ( 1.7) | 15 ( 9.0) | <0.001* |
|  | Grade 1 | 58 (12.8) | 21 ( 7.3) | 37 ( 22.3) |  |
|  | Grade 2 | 105 (23.1) | 56 ( 19.4) | 49 ( 29.5) |  |
|  | Grade 3 | 107 (23.6) | 70 ( 24.3) | 37 ( 22.3) |  |
|  | Grade 4 | 107 (23.6) | 83 ( 28.8) | 24 ( 14.5) |  |
|  | Grade 5 | 57 (12.6) | 53 ( 18.4) | 4 ( 2.4) |  |
| Left Lower limb muscle strength grading（proximal ） | Grade 0 | 16 ( 3.5) | 3 ( 1.0) | 13 ( 7.8) | <0.001* |
|  | Grade 1 | 38 ( 8.4) | 10 ( 3.5) | 28 ( 16.9) |  |
|  | Grade 2 | 51 (11.2) | 31 ( 10.8) | 20 ( 12.0) |  |
|  | Grade 3 | 84 (18.5) | 49 ( 17.0) | 35 ( 21.1) |  |
|  | Grade 4 | 169 (37.2) | 121 ( 42.0) | 48 ( 28.9) |  |
|  | Grade 5 | 96 (21.1) | 74 ( 25.7) | 22 ( 13.3) |  |
| Left Lower limb muscle strength grading（distal） | Grade 0 | 22 ( 4.8) | 8 ( 2.8) | 14 ( 8.4) | <0.001* |
|  | Grade 1 | 39 ( 8.6) | 13 ( 4.5) | 26 ( 15.7) |  |
|  | Grade 2 | 46 (10.1) | 25 ( 8.7) | 21 ( 12.7) |  |
|  | Grade 3 | 88 (19.4) | 53 ( 18.4) | 35 ( 21.1) |  |
|  | Grade 4 | 153 (33.7) | 106 ( 36.8) | 47 ( 28.3) |  |
|  | Grade 5 | 106 (23.3) | 83 ( 28.8) | 23 ( 13.9) |  |
| R_APB |  | 2.09 (2.57) | 2.48 (2.82) | 1.57 (2.10) | 0.024* |
| R_ADM |  | 2.96 (2.79) | 3.49 (2.89) | 2.23 (2.49) | <0.001* |
| R_FDI |  | 2.26 (2.91) | 2.65 (3.05) | 1.76 (2.64) | 0.003* |
| L_EDB |  | 2.23 (2.59) | 2.73 (2.79) | 1.55 (2.12) | <0.001* |
| L_ADM |  | 2.71 (2.55) | 3.12 (2.50) | 2.13 (2.52) | <0.001* |
| L_FDI |  | 2.16 (2.79) | 2.62 (2.91) | 1.54 (2.50) | <0.001* |
| R_EDB |  | 2.58 (2.23) | 3.07 (2.25) | 1.93 (2.03) | <0.001* |
| R_AH |  | 8.65 (5.15) | 9.43 (4.87) | 7.59 (5.35) | <0.001* |
| L_EDB |  | 2.28 (2.06) | 2.81 (2.18) | 1.57 (1.63) | <0.001* |
| L_AH |  | 8.34 (5.08) | 9.06 (4.79) | 7.38 (5.33) | 0.011* |

Note: R_APB: Right Abductor Pollicis Brevis (median nerve); R_ADM: Right Abductor Digiti Minimi (ulnar nerve); R_FDI: Right First Dorsal Interosseous (ulnar nerve); L_EDB: Left Extensor Digitorum Brevis (common peroneal nerve); L_ADM: Left Abductor Digiti Minimi (ulnar nerve); L_FDI: Left First Dorsal Interosseous (ulnar nerve); R_EDB: Right Extensor Digitorum Brevis (common peroneal nerve); R_AH: Right Abductor Hallucis (tibial nerve); L_EDB: Left Extensor Digitorum Brevis (common peroneal nerve); L_AH: Left Abductor Hallucis (tibial nerve).
