# Supplementary figures and images for: Imaging-derived neuromuscular ultrasound phenotypes are associated with functional status in amyotrophic lateral sclerosis
Source: J Neurol. 2026 Feb 21;273(2):158. doi: 10.1007/s00415-026-13705-4 (PMC12924791; doi:10.1007/s00415-026-13705-4)

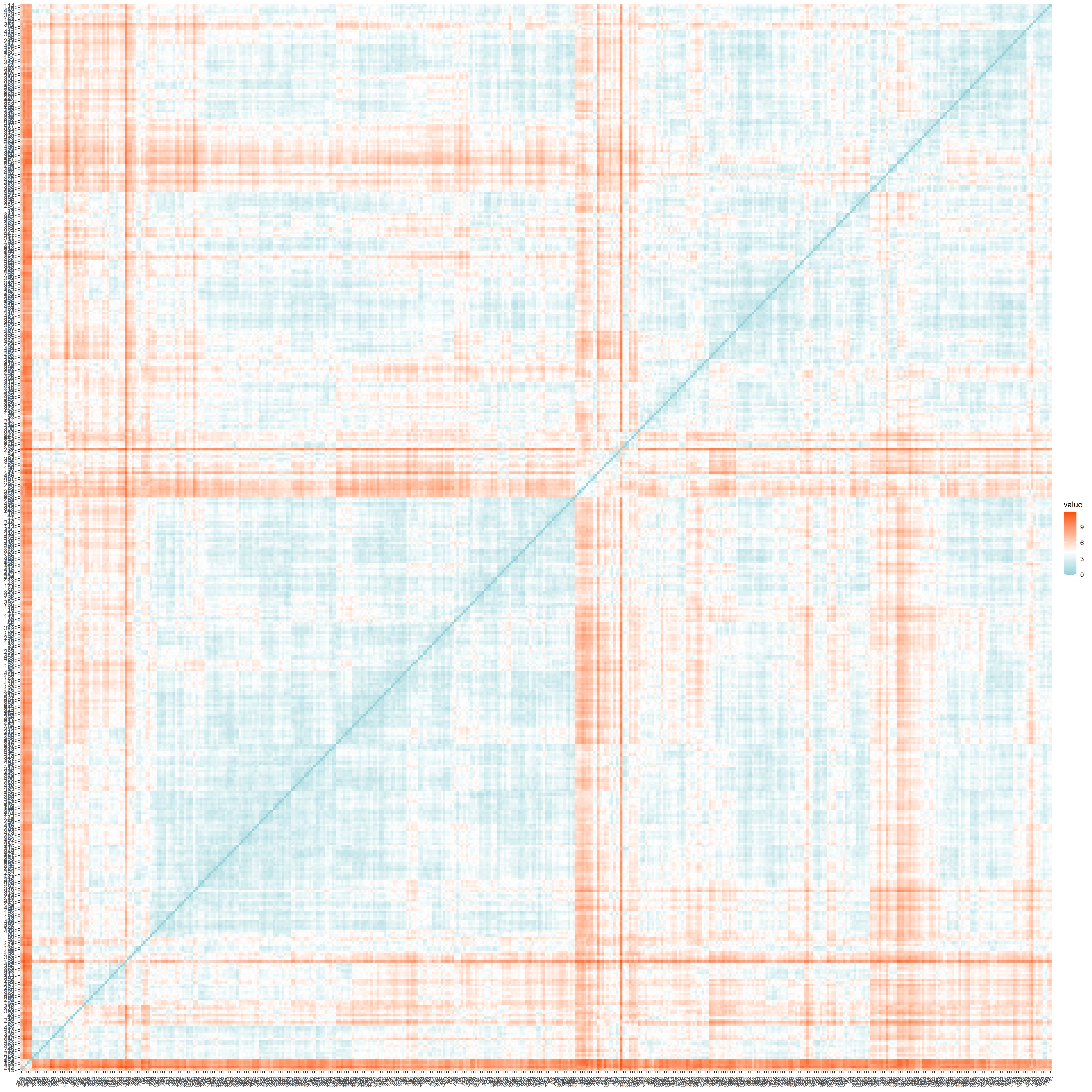

Supplement: Supplementary file 3 — Supplementary file3Figure S1. Pairwise distance matrix heatmap of the clustering feature space. A heatmap of the pairwise distance matrix computed from the standardized clustering feature set (NMUS variables). Each row/column represents one participant, and each cell indicates the distance between a pair of participants (cool colors, smaller distances/more similar profiles; warm colors, larger distances/more dissimilar profiles). The diagonal corresponds to self-distance (zero). Apparent block-like patterns reflect subgroups of participants with relatively similar multivariate ultrasound profiles. This visualization is provided to illustrate the overall structure of the feature space and was not used as a criterion to determine cluster number (JPEG 7672 KB) [file 415_2026_13705_MOESM3_ESM.jpeg]

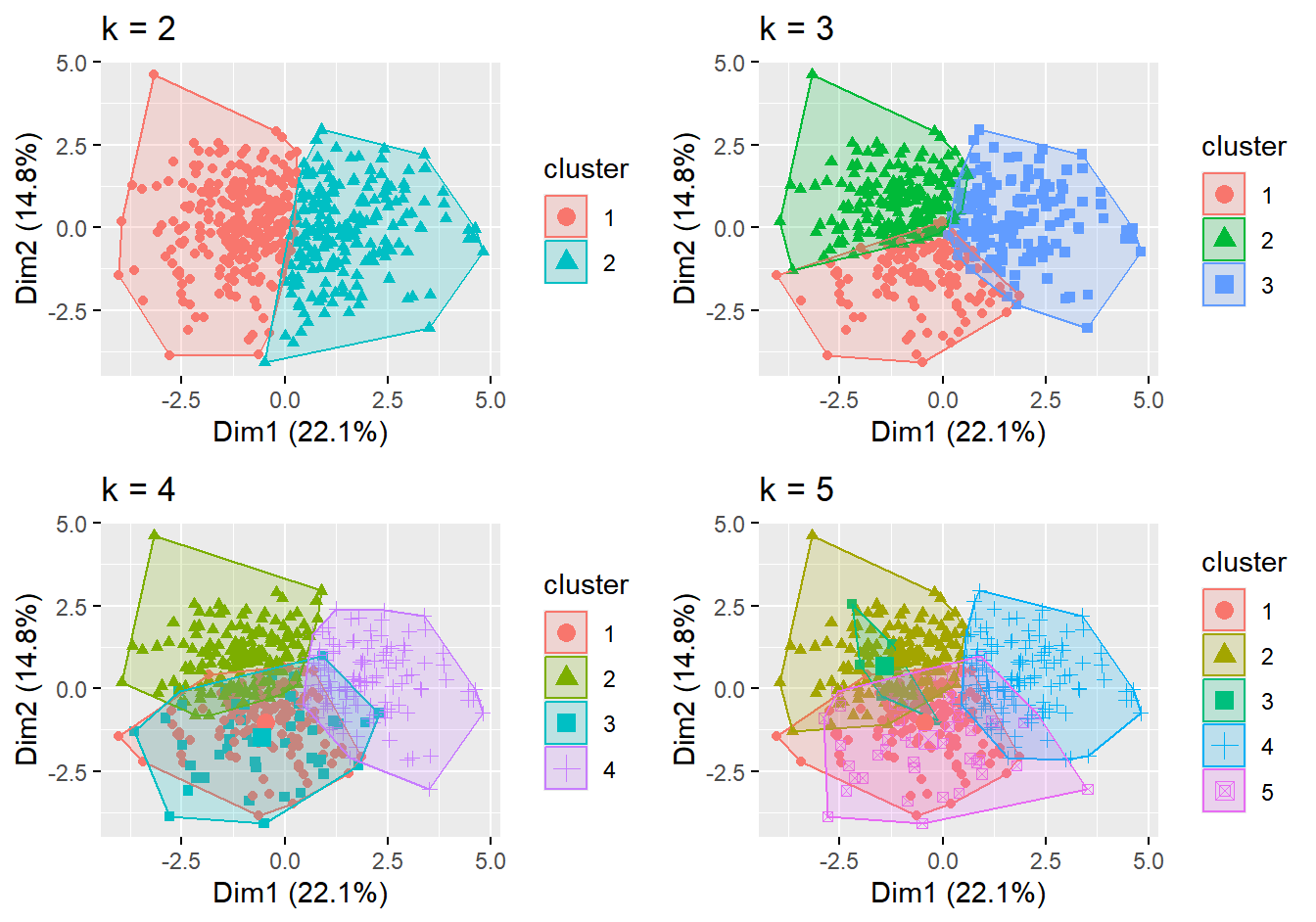

Supplement: Supplementary file 4 — Supplementary file4Figure S2. Cluster separation across different numbers of clusters (k = 2–5). Two-dimensional projection of participants based on the NMUS clustering features, visualized under different k values (k = 2, 3, 4, and 5). Points represent individual participants and are colored by their assigned cluster at each k. Shaded polygons indicate the approximate convex hull (cluster boundary) of each cluster in the projected space. The displayed axes (Dim1 and Dim2) correspond to the first two dimensions of the visualization space (e.g., principal components or a similar linear projection used for display), and the percentage values indicate the proportion of variance explained by each dimension. This figure is provided as a supplementary visualization to compare the apparent separation and overlap of clusters under alternative k settings, whereas the primary selection of k in the main text was based on the pre-specified criterion (e.g., silhouette optimization) (PNG 119 KB) [file 415_2026_13705_MOESM4_ESM.png]

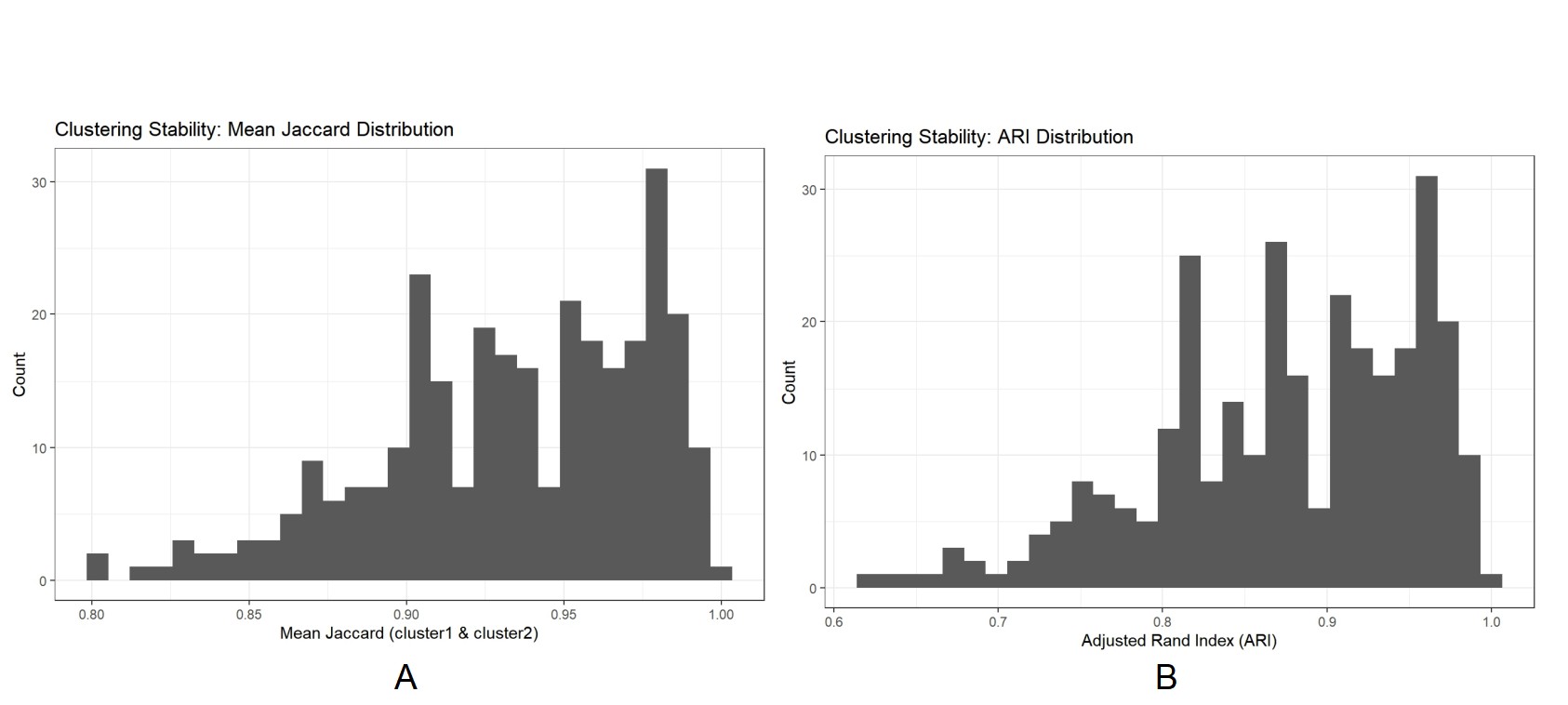

Supplement: Supplementary file 5 — Supplementary file5Figure S3. Resampling-based stability of the ultrasound-derived two-cluster solution. (A) Histogram of the mean Jaccard similarity (averaged across the two clusters) comparing cluster membership from each resampling run with the original k = 2 reference clustering. (B) Histogram of the adjusted Rand index (ARI) comparing resampling-based cluster assignments with the original k = 2 solution (JPG 89 KB) [file 415_2026_13705_MOESM5_ESM.jpg]

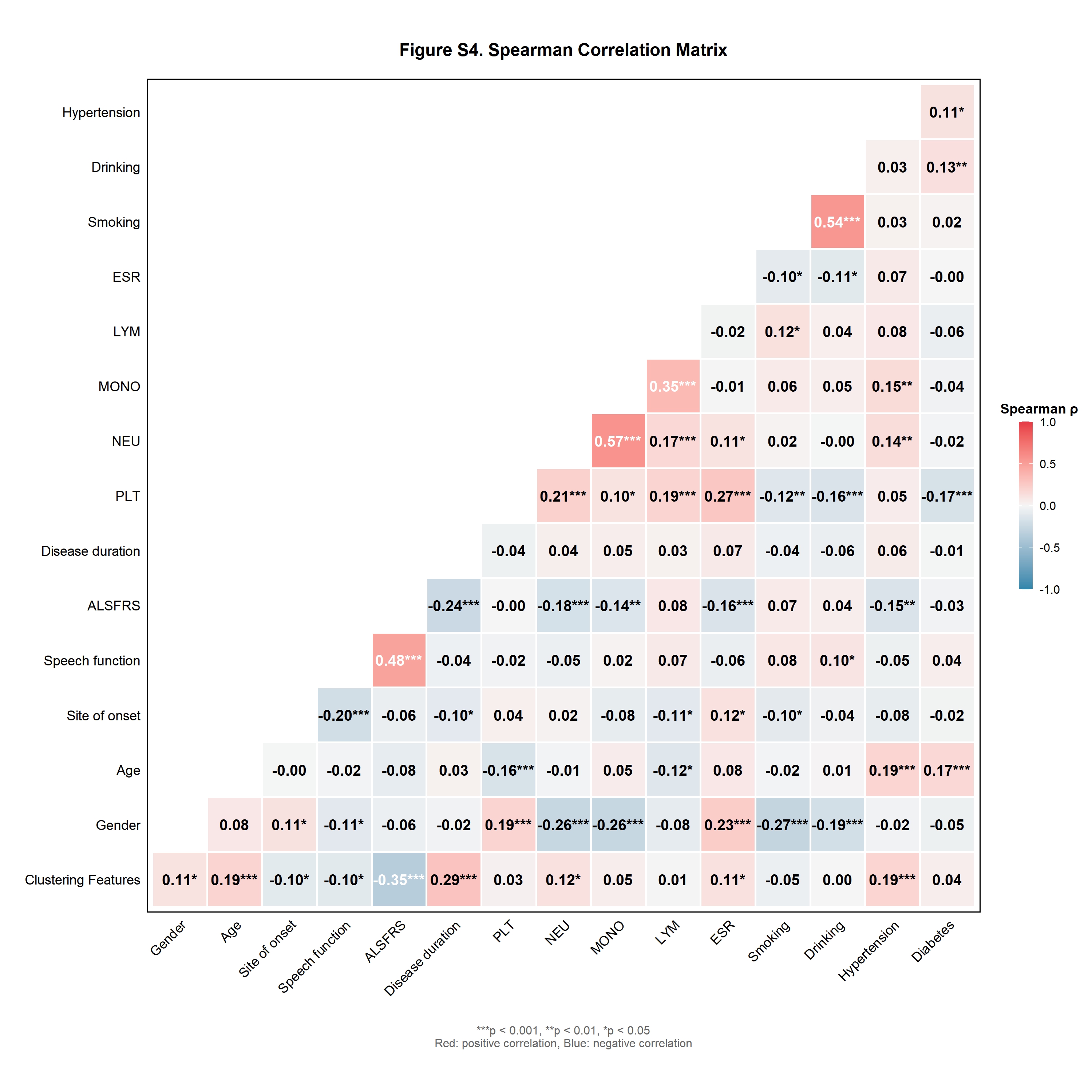

Supplement: Supplementary file 6 — Supplementary file6Figure S4. Spearman correlation matrix of clinical characteristics, inflammatory markers, and cluster-defining features. Spearman correlation matrix across clinical characteristics (e.g., demographics and disease-related variables), inflammatory markers, and cluster-defining features. Colors represent Spearman’s ρ (red, positive; blue, negative). The diagonal indicates self-correlations. Significance is annotated as *P < 0.05, **P < 0.01, and ***P < 0.001 (two-sided) (JPEG 1308 KB) [file 415_2026_13705_MOESM6_ESM.jpeg]
