# Supplementary material for: Imaging-derived neuromuscular ultrasound phenotypes are associated with functional status in amyotrophic lateral sclerosis
Source: J Neurol. 2026 Feb 21;273(2):158. doi: 10.1007/s00415-026-13705-4 (PMC12924791; doi:10.1007/s00415-026-13705-4)
Supplement: Supplementary file 7 — Supplementary file7 (DOCX 14 KB) [file 415_2026_13705_MOESM7_ESM.docx]

**Supplementary Table S3. Reliability of ultrasound measurements.**

| **Variables** | **Number of subjects** | **ICC** | **95% CI** | **SEM** | **MDC95** |
| --- | --- | --- | --- | --- | --- |
| **US‑TMT** | 30 | 0.911 | 0.864–0.930 | 0.191 | 0.529 |
| **US‑TMM** | 30 | 0.944 | 0.913–0.957 | 0.1 | 0.278 |
| **US‑BT** | 30 | 0.94 | 0.907–0.953 | 0.127 | 0.351 |
| **UI‑BH** | 30 | 0.947 | 0.918–0.959 | 6.836 | 18.949 |
| **US‑TFIDM** | 30 | 0.914 | 0.870–0.933 | 0.059 | 0.164 |
| **UI‑FDI** | 30 | 0.891 | 0.836–0.915 | 12.643 | 35.046 |
| **US‑RFRMT** | 30 | 0.932 | 0.896–0.947 | 0.123 | 0.342 |
| **UI‑RRFH** | 30 | 0.933 | 0.897–0.948 | 7.614 | 21.104 |
| **MNCSA** | 30 | 0.88 | 0.821–0.906 | 0.009 | 0.026 |
| **UNCSA** | 30 | 0.878 | 0.818–0.904 | 0.01 | 0.028 |

Inter-operator reliability for ultrasound measurements in 30 participants. ICC denotes the intraclass correlation coefficient based on a two-way random-effects model for absolute agreement using single measurements. SEM denotes the standard error of measurement and MDC95 denotes the minimal detectable change at the 95% confidence level. Number of subjects indicates the number of paired measurements used for each estimate. SEM and MDC95 are reported in the same units as the original measurements.
